# On German Psychology

**Published:** 1848-10-01

**Authors:** 


Art. II.—*The Principles of Medical Psychology; being the Outlines of a Course of Lectures.* By Baron Ernst Von Feuchtersleben, M.D. Translated from the German by the late H. Evans Lloyd, Esq. Revised and edited by B. G. Babington, M.D., F.R.S., &c.

(*Second Notice.*)

We proceed, in accordance with our promise, with the analysis of the concluding portion of Baron Ernst Von Feuchtersleben's volume, in which he treats of the therapeutics of the subject, and of judicial psychology. In reference to the therapeutical department, we shall follow our author's arrangement, and first consider the exclusively psychical mode of treatment, then examine the effect and application of physical remedies to psychical conditions, next proceed to those of both kinds, and, after we have done this, we shall review the therapeutics of mental disorders, according to their several symptoms.

In the purely psychical mode of cure it must be recollected that "the vehicle, as it were, in which the medicine is exhibited, is the person of the administering physician himself." Hence, in this mode of treatment, everything depends upon him; and, on this account, it doubtless is, that many writers, in describing the character of the psychopathic physician as it ought to be, have set up an unapproachable ideal standard, which, strictly speaking, could be reached by no one. We have already (see p. 248) noticed those qualifications which are peculiarly requisite to the psychological physician, and shall therefore proceed to the consideration of psychical remedies.

We commence with remedies applied through the senses. These operate through the *sensorium commune* upon psychical action, first affecting attention, and through it, imagination and memory, and finally influencing the power of thought.

The touch is too remote from the mind to be made very available. The taste is more readily applicable. A delicacy in which he formerly rejoiced will put a hypochondriacal epicure into comparatively good spirits, while, on the other hand, nauseating substances usually divert the attention of the lunatic from things around him, and fix it on himself.

Our author considers that the smell has not yet been made sufficiently available, and suggests (as indeed was proposed by Reil) that a casket of perfumes would afford an object for the exercise of attention; he hints also at the further importance of the employment of this sense on account of its special relation to memory.

The sight is well adapted to influence the mind. There can be no doubt that light, darkness, and colour, exercise very decisive psychical influences; while light acts as an excitant, and twilight exerts a calmative influence, darkness, according to circumstances, acts in either manner. The positive colours are asserted to act as stimulants, and the negative as sedatives. In our former article we noticed the fact that Aretæus laid great stress on the colour of the rooms inhabited by psychical patients. Rösch and Esquirol affirm from observation that indigo dyers become melancholy; and those who dye scarlet, choleric. Their observation regarding indigo dyers affords a strong confirmation of the statement of that arch-quack, Paracelsus, who declared blue to be injurious. Feuchtersleben suggests that, as the psychical effect produced by coloured glasses is particularly strong, it might be desirable to let certain patients, with morbid ideas, see the world through stained windows or coloured spectacles, with the green hue of hope, or the red glare of pomp.

With reference to the sense of hearing as a means by which we may act on the mind, our readers will recollect that we have already noticed one or two cases in which beneficial results followed the use of music. Our author quotes a case (from Reil) in which a lunatic was lulled to sleep and cured by the noise of water dropping from the ceiling of his room into a copper vessel. Reil's cat harpsichord, in his opinion, is more likely to cause madness than to cure it. We fully concur with him in this view, and so doubtless will our readers when we inform them how cat harpsichords are constructed. "Cats, chosen according to the musical scale, are placed in a row with their tails turned outwards; the keys furnished with sharp nails fall on their tails, and the cat which is struck squalls forth its note."

Feuchtersleben is of opinion, and, we think, correctly, that music as a remedy has been far too much neglected in modern practice, and he adds that for therapeutic purposes in these cases more is to be expected from simple, melodious music, which speaks directly to the feelings, than from that which is artificial, founded on the laws of harmony, and addressing the feelings through a cultivated knowledge of the art. In deciding, however, on the kind of music to be employed, much must depend on the taste and special knowledge of the patient.

We proceed to the consideration of remedies applied through the attention. These may be arranged as under:—

1. Diversion.
2. Concentration of attention: and—
3. The awakening of obscure ideas, and repressing such as are too predominant.

We shall offer a few remarks on these separate heads.

The means that may be adopted, in cases of profound mental absorption, to rouse and divert the thoughts, are well known, and need not be particularised. They must be derived from, and adapted to, the individual circumstances of the case.

We extract the following maxims to be observed in their application:—

"*a.* The remedy employed for dissipating the thoughts must correspond in quality with the course of ideas of the patient. Extremes act only for the moment upon one another; durable effects require gradual transitions.

"*b.* With respect to quantity, it must correspond in strength with the morbid ideas of the patient.

"*c.* It must apparently be accidental: if the patient be aware of design, the effect ceases. Hence it is so difficult to dissipate one's own thoughts.

"*d.* Care must be taken that the dissipation do not lead to an abnormal new direction of the mind, to which there was a predisposition.

"*e.* The dissipating remedy must be varied, but not too suddenly.

"*f.* The treatment must be continued for a sufficient length of time, because the old ideas are so apt to return."—pp. 323, 324.

Proceeding to the second head, we find our author laying all due and proper importance on order and employment, as the most important psychical remedies for vague thinking. We extract his remarks on these two points:—

"*a.* Order; the form of healthy activity is gradually followed by its reality. The psychical patient who has become accustomed to rise at the same hour, to wash and dress himself, and to follow the routine of the day in taking his meals and sleeping, has taken the first step towards recovery.

"*b.* Employment, which must be suitable to the circumstances of the individual, and not too uniform, is the second *conditio sine qua non* of psychical cure. It must not be merely mechanical, or pursued in the form of treatment, but to the patient it must seem to have an external object. It must be varied according to circumstances, from hard corporeal labour, as agriculture and gardening, to mechanical, as working in pasteboard and basket-making, and from this to intellectual employment, as reading aloud, dictating, writing exercises, making extracts, translations, &c. (not, however, knitting, sewing, &c., which, from the uniformity of their mechanism, give too little occupation to the mind, and rather facilitate its reveries.) Employment acts both physically and ethically, inasmuch as it teaches the wandering mind to return from its visionary world to the common daily duties of human life."—pp. 324, 325.

Under the third head—the awakening of obscure ideas and repressing such as are too predominant—our author observes very truly that the former object is effected "partly by employment on objects in which the patient formerly took particular interest, partly by calling into action his latent powers;" while the latter is effected "in part, indirectly by the same process, in part directly in a negative way, by paying no attention to them, which, in most cases, is better than attempting to refute them." The patient may in this way often be able to contribute very materially to his own cure. A hypochondriac, attended by Feuchtersleben, became convalescent from the moment that he was prohibited from continuing a journal which he kept of his condition.

The observations on the means for dissipating thought conclude with the remark that smoking, for those who are used to it, deserves particular notice as a means of recreation. Although, as a general rule, we are opposed to the habit of tobacco-smoking, we are able from personal observation to testify to its occasional good effects. "The tobacco-pipe," says an eminent German psychopathic physician, "follows tranquillity of mind as the latter does the tobacco-pipe."

Remedies may be applied through the memory. Here we endeavour to strengthen a dull memory by exercising it in learning by rote—an exercise by which we attain the advantage not only of strengthening the memory, which, in many cases, as for instance in idiocy, constitutes an essential part of the cure, but also of diverting the attention in cases of fixed delusion, and of fixing it on another object in cases of distraction. The subject selected should be always one that interests the feelings. Learning a foreign language is one of the best exercises of this nature.

In the consideration of these psychical remedies we must not omit to notice the influence of the fine arts on patients who are susceptible of being affected by them. In this point of view theatrical representations are often highly serviceable, since they combine all the effects of art—poetry in the composition; painting in the scenery and acting; and music in the overtures and interludes. It must, however, be recollected, that such representations are powerful both for evil and for good, and that the greatest caution is requisite on the part of the physician in deciding on the cases in which they are likely to prove serviceable. The reports of many of our British asylums testify to the value of these exhibitions. Popular lectures on chemistry and natural philosophy, illustrated by striking experiments, constitute an excellent employment for the mind, and have been spoken of in high terms by British physicians: on this subject our author makes no remarks. We extract the following remarks on the importance of attending to the general improvement and employment of the mind :—

"Culture is one of the surest protections against the inroads of insanity, and in this respect, psychical treatment bears a great resemblance to education, with which (under the term 'psychagogics') it is so often compared. But here, again, everything must be adapted to individuals and to circumstances, and the appearance of design and dictation must, as far as possible, be avoided. Of all the scientific employments which may here be made available for our purpose, mathematics have, undoubtedly, the preference. They alone afford that complete evidence and conclusiveness which satisfy the requirements of man's reasoning powers. They arrange and exercise them, as it were, unconsciously; they are difficult enough effectually to abstract the mind from other trains of thought, and leave fancy and passion undisturbed. Even logic, if the study of it were admissible, does not afford these advantages, because, when half studied, it only serves to support the sophistry of delusion, and forms points of transition to the dangerous abstraction of metaphysics. Hoffbauer tells us of a lawyer, who having, when in the deepest distress, applied himself to the study of mathematics, succeeded in raising himself from a state of despair."—p. 328.

In those cases in which the patient does not take genially to his proposed mathematical studies, (and we should conceive that even in Germany such cases are not very rare,) he is allowed to take up the physical sciences; but when the mind is closed against all these remedies—when the mathematics and natural philosophy delight him not, and he rejoiceth not in music (which, according to our author, may very well be united with mathematics)—there is still a welcome resource left in cards, backgammon, and chess.

Under the consideration of remedies acting through the feelings, we must notice the subject of rewards and punishments. Our author very justly remarks that there is sense in rewards and punishments, only so long as the patient can be improved by them; when improvement is unattainable they lose their object, and measures of security should take their place.

In the application of rewards and punishments the following precautions should be observed:—

"1. The motives of reward and punishment must counterbalance in energy (i. e., in degree) the motives of the delusion. Half measures must be carefully avoided, as they only produce a stronger reaction. Drunkards, in particular, require strict treatment.

"2. We should be severe towards the faults of the patient, mild towards his person. We ought, therefore, to be just, equitable, and, above all, perfectly dispassionate. Punishment with anger excites a bitter feeling, and wholly fails of its object. How can the patient receive it as a remedy applied by a physician who desires his welfare? To impress his mind with the idea that justice is done him, is the business of the medical man, and it is not always an easy task. The pure notion of right is by no means to be taken here as the guide, for it is not applicable to the mentally diseased patient. The degree of this applicability must be measured according to the actual state of the consciousness of the patient—that is, according to the degree of freedom in his volition, and to the object of the punishment in the particular case.

"3. The punishment, as well as the reward, must immediately follow its cause, and be continued so long as the patient has the consciousness of its applicability to his conduct.

"4. Regard must be had to the milder or rougher dispositions, and to the character and station of the patient, that we may avoid committing faults or absurdities.

"5. We must be more prompt and liberal in the bestowal of rewards than of punishment, in order constantly to remind the patient of the intention to effect his cure.

"An adherence to these maxims constitutes, practically, the difference between discipline and mere drilling, the latter of which presupposes no consciousness, and is therefore, at most, applicable only in the lowest psycho-physical condition in the cure of idiots of the more advanced degree, or in the most furious attacks of mania (*pro momento*), where the patient must, like an unconscious natural power, be prevented from injuring himself and others."—p. 329.

Amongst the most appropriate punishments our author places a partial deprivation of food, a prohibition from walking out, from visiting the garden, or from greater liberty in the case of more docile individuals; a deprivation of books, a denial of some refined amusement, as, for instance, music, among the more educated patients; whilst there are cases in which a disapproving silence or a reproachful look is found to act with the necessary power. We are glad to see that he is decidedly opposed to the system of painful corporeal chastisements—a barbarous system, that seems to have forced its way, during the dark ages of psychology, into the purgatories for the reception of the insane in all countries. It is little more than half a century ago since Lichtenberg enunciated his celebrated aphorism (which, by the way, our author refers to in a foot note) that "The mind of fools is obliged by a cudgelling to remember the world from which the cudgel comes."

The psychical effects of the various feelings—of self-approbation, religious feeling, hope, fear, and honour, vexation and shame—are duly considered; and we then arrive at the subject of corporeal remedies considered in reference to their psychical action. They may be regarded as having a threefold relation—

"1. To the mental action itself.

"2. To those diseases of the body which stand to the psychopathies in the relation of causes.

"3. To those diseases which accompany the psychopathies as complications."—p. 334.

We have already incidentally noticed the first series of remedies, in page 501: we shall therefore only enumerate some that have not been previously mentioned.

"*a.* Exercise, either active—as mechanical, agricultural, or horticultural labour, gymnastics, riding, walking, skating, swimming, dancing, &c.; or passive, as swinging, driving in a carriage, &c."

In reference to the subject of travelling, we suspect our author holds much the same views as Dr. Seymour. (See p. 9, and his "Thoughts on the Nature and Treatment of several severe Diseases of the Human Body," vol. i. p. 179.) It "works miracles, but, it must be confessed, *only in milder cases.*" Feuchtersleben considers that the advantages derived from frequenting watering-places arise, in part, from the circumstance that the patient is compelled, from want of convenience, to take care of himself.

"*b.* Lowering treatment of all kinds—hunger, thirst, blood-letting, purgation, cold, deprivation of sleep."

These, according to our author, act psychically by weakening the energy of the mental action when it is excited. We regret that he has not given cases illustrative of the advantages derived from thirst and deprivation of sleep.

"*c.* Derivative and alterative remedies—as shower-baths, counter-irritants (blisters, setons, moxas, the inunction of tartar emetic ointment, &c.); also, calomel, carried to salivation, and tartar emetic in full or divided doses. These act psychically by drawing off the attention which is improperly fixed, and at the same time, according to circumstances, exciting a lowering mental activity.

"*d.* Stimulants—wine, opium, warmth, friction, electricity—act psychically, by exciting a feeling of unrestrained organic vital energy. They must be used with moderation and a strict attention to the individual case."

In regard to opium and narcotics generally, our author observes, that, belonging to the class of stimulants, they must be used as such, with very great precaution, on account of their dangerous after-effects, so that pure stimulants should be substituted for them.

"In their total effect—that of causing stupefaction—they are almost absolutely contra-indicated, as respects our object; they always diminish the efficacy of the psychical principle; if too long continued, they change every form of psychosis into almost incurable idiocy; and they may, in their tranquillising power, be advantageously replaced by other sedatives."—p. 336.

We have in our own experience seen so much good from the prolonged use of opiates in cases of melancholia, that we cannot help suspecting that our author speaks theoretically rather than practically on this point.

We proceed to the consideration of a subject, which, from its openness to deception and from the numerous impositions that beyond all question have been practised regarding it, has hitherto, except amongst a certain class who can hardly be said to enjoy the confidence of the profession at large, failed to excite the attention which as a therapeutic agent it fairly demands. Need we add that we refer to animal magnetism? We shall not enter into its theory, nor yet on the mode of practising it. We shall confine ourselves to its therapeutic value. It is doubtless a remedy of much power and of considerable danger, if injudiciously applied. One psycho-physical state of disturbance is, as our author observes, purposely opposed to another, perhaps more dangerous one, already existing; in the same manner as with reference to the body, we oppose to certain cachexies a mercurial or iodine disease—an experiment which it is evident is always an affair of conscience and an individual problem for the solution of the physician.

Our author is of opinion that the so-called animal magnetism may be employed as a remedy with a threefold object:—

" 1. To tranquillise for this purpose, simple magnetic sleep is sufficient —nay, often (as for instance in toothache, slight convulsions, &c.,) tractation suffices, without the induction of sleep. This harmless manipulation may at any rate be sometimes tried, and may have an advantageous effect, psychically, through the feeling of hope arising from confidence in its efficacy; and physically, through centripetal enervation.

" 2. By a higher degree of vitality to excite, through the nervous system, a salutary (metasyncritical) re-action. This procedure demands great caution and individual discrimination, since, after all, it is only proceeding by guess. The physician may here be compared to one attacking the case with a stick. If he strike the disorder, so much the better, if he strike the patient, so much the worse. The question always is, how far may a psycho-physical exaltation go in *this* or *that* individual patient, in order to produce only a *salutary* excitement, and where lies the point at which this excitement may become *dangerous?* It can never be a matter of indifference; he, therefore, who will venture to give a decided answer to this question, as applied to the case which he is treating, may likewise venture on this procedure.

" 3. To obtain, by the so-called *clairvoyance*, prescriptions from the patient himself as to the treatment of his case. He who has rightly conceived the essence of magnetism to be an over-wrought dream with extraneous psychical influence, may consider, in the individual case which he is treating, whether he can truly expect, from the heightened instinct of his patient, a better insight into the case than from his own scientific knowledge. When the latter leaves him wholly at a loss, and nothing is risked, he may venture on the experiment.

" It is sufficiently evident, from what has been stated, that merely making experiments with magnetism to gratify scientific curiosity, is cruel; and to gratify philosophic or religious curiosity, is foolish. This is not employing it as a remedy.

" The so-called magnetic treatment thus offers, physically, a calming or enervating remedy, and psychically, an experiment which excites the fancy. It acts as a mixed method through a mean state between psycho-physical health and disease, and should, therefore, at most, be tried only in such mean states.

"In what the efficacy of this strange method properly consists, has not yet been decided by science. At all events, the term magnetism, arising out of Mesmer's history and hypothesis, is wholly gratuitous, provisional, and by way of analogy. The term "mesmerism," which is here and there used, though the moderns differ much from Mesmer, would still, therefore, be more suitable. We are by no means informed how the forces of telluric, or mineral magnetism, which, with those of the metallic tractor—the so-called rhabdomancy—require a more accurate physical elucidation, are related to these psycho-physical phenomena. To deduce them, as sometimes has been done, solely from the focus of generation, and consider them as merely hysterical, though the sexual developments have certainly much to do with them, would be to take a partial and, I should say, unjust view, considering the character of many physicians known to us as friends of this method, and the experiments which we ourselves have occasionally had the opportunity of making on wholly unprejudiced young boys. To explain magnetism by the "rapports" in which all natural beings stand to each other, as was done formerly by F. Hufeland, and more recently by Ennemoser, is evidently extending the ground of explanation too far, as the question here has reference to a more definite support. It is commendable in Ennemoser, that while he extols the improvement of somnambulists by magnetism, he likewise notices the fact of their being made worse by it, and it is certain, as Eschenmayer has already observed, that the final explanation of this much talked of and often abused mode of cure, is to be hoped for only by pursuing the course which we take in our inquiries between psychology and medicine—namely, pathology; but not even thus, until some one possessing the requisite qualities shall be found to devote himself to the subject."—pp. 338—340.

Our author is evidently a waverer in his belief. There is obviously a gentle spirit of irony in the observations on *clairvoyance*, and yet he feels much as we all feel but do not all dare to confess, that there is beyond all question *a certain amount of truth* in this so-called animal magnetism. There are, he adds, many reports of experiments of this mode of cure which bear the stamp of faithful observations; for instance, those of Dr. Spiritus, in Nasse's Zeitschrift, 1822, vol. i. &c. "*Experiments of this kind, which are to be mentioned elsewhere, have been adduced by myself, which forbid me to express a positive judgment in the negative.*"

We now come to the treatment of the different forms of psychopathies. Our author lays great stress on the maxim, that in insanity more than in every other affection, we should treat the patient more than the disease: no two persons being alike, each must be treated on its own merits. In this, doubtless, all our readers will agree with him. The remedial dietetics of the mind (acting as prophylactics) then claim his attention. Much matter of high interest meets us in this portion of the volume, and we regret that our allotted limits warn us from extracting more than the following brief quotation:—

"Langermann, if not the first, was at all events the foremost to compare the treatment of mental disorders to education, and to advise mental development and cultivation with a therapeutical view. As a prophylactic, it is fully adequate to its object as a remedy, and that the best, the comparison fails—the difference between the education of youth and psychical treatment must be the same as that which exists between their objects—childhood and insanity. Children and lunatics are said to have this in common—that they speak the truth; they have many other and more important resemblances, but likewise very many differences, both will be easily discerned by the attentive observer. Moreover, that self-command is possible even in a state of mental disease, and consequently offers a means of cure, is proved by the cunning of lunatics, by which they are often able for a long time to conceal their insanity.

"We will add here one special remark, that females in particular, with a decided predisposition to mental disorders, are often, as by a sure prophylactic, preserved from them in the harmonious exercise of their psycho-physical life by the regulating influence of marriage (we mean a comparatively happy marriage of course)."—p. 343.

We proceed to the consideration of the treatment to the leading form of the psychopathies:

"Folly," says our author, "offers no small difficulties in its treatment. A command over the patient must constitute the commencement of the cure. He must be brought into subjection by coercive measures, circumstances must be changed, and new ones substituted, the main principle of these must be order, to which the confused psychical actions of the patient must be habituated. Occupation is here the chief psychical remedy, in order to arrest the vague flight of ideas. The coercion, however, must not be violent and continued, but must be mitigated or increased, as a reward or a punishment, according to behaviour. The kind and degree of employment from field-labour to reading must be suited to the ability and inclinations of the patient, his fancy must on no account be consulted. An object must always be held out which shall not appear as a mere remedy; the relation of the exercise to the understanding must be closely in correspondence with the returning activity of the latter, and direct correction of its irregularity must not take place till there is the decided improvement.

"Suitable psychical measures must be opposed to the several psychical symptoms: to absence of mind, a fixation of the attention to one object of interest; to forgetfulness, an exercise of the memory; to talkativeness, solitude, which affords no opportunities for conversation; to bursts of passion, a categorical, imperative; to the present morbid inclinations of the patient, his former anamnestically known ones; a generally suitable discipline with respect to instruction is above all things indicated in this form."—p. 344.

On the somatic side there are various symptoms—nervous erethism, decided periodicity, spasms, and hallucinations. The danger of these symptoms must be counteracted by nervine remedies, especially by tartar-emetic in increasing doses from two to eight grains, in six ounces of distilled water. Our author expresses himself as strongly opposed to the unction of tartar emetic ointment and issues in these cases. This is a point on which, we conceive, no general rule can be laid down.

Fixed delusion is the next psychopathy whose treatment claims our attention. We extract the remarks on the mode of cure in cases of religious fixed delusion, erotic fixed delusion, and melancholy:

"In religious fixed delusion, accompanied with remorse, the religious feeling itself must be employed as a cure, by endeavouring to change its direction, and by inspiring confidence in the infinite mercy of God. In religious fixed delusion, combined with ecstasy, occupation of the soberest kind, alternated with cheerful recreation, must aid the direct instruction which may be practicable, and which must always be founded on the idea of religion.

"In the erotic fixed delusion, occupation is likewise an indispensable condition. The advice which even Reil gave to gratify the longing which is here the foundation of the disease, would, if followed, almost certainly lead to destruction. Let us reflect that here again it is not the individual idea, but that which fixes it, which is the point in question, and that the gratification of the desire never cures, but, on the contrary, increases it. A diversion to another object of the same desire, and a change of this diversion, would be more likely to do good, if it were not so difficult to effect it. It is to be observed, moreover, that here, in every case, physical remedies, especially the antiphlogistic method, must likewise be resorted to in order to lower the sexual orgasm.

"In melancholy, the cure by diversion, but with caution and strict regard to the state of the individual, is applicable in its greatest extent. If it assume the character of spleen, sense of honour, duty, and religion, are perhaps the only interest which can rouse the deadened vitality of the mind. If it assume the character of thanatophobia, let the cure by means of occupation, gymnastics, riding, &c., be declared to be a cure for the disorder of which the patient is afraid he shall die. Emotions, sometimes of the sthenic kind, but often asthenic—*e. g.*, fear, which furnishes a negative excitement, are likewise useful in every kind of melancholy."—pp. 347, 348.

Somatically the fixed delusion is often referrible to abdominal disorder. The irritation of the mucous membrane of the bowels, which extends to the abdominal nervous plexus, and from thence to the sensorium, may arise from various causes, and must be treated accordingly. Laxatives, derivatives, purgatives, warm baths, and certain mineral waters, especially those of Carlsbad, are of service in these cases. We have already mentioned that melancholy is often associated with and apparently dependent on cardiac disease. A combined antiphlogistic and sedative treatment is here indicated, but these cases often present great difficulty to the physician.

We proceed to the consideration of the treatment of mania, which, although the most curable of the psychopathies, demands the most energetic mode of cure.

The primary difficulty in the treatment of mania consists in determining in individual cases whether the excess of excitement shall be checked or not, and if checked, in what degree. While, on the one hand, it appears natural to let the excitement take its course and exhaust itself, we must, on the other hand, recollect that the frequent repetition will render the impression on the motor system more permanent.

"This consideration," says our author, "leads to three principal rules of treatment:—1. The patient must be tamed, that he may not injure himself and others. So far, therefore, the constraint, which is not to be looked upon as a cure, but as a measure of police, must be carried in every case. 2. The patient must be treated in a twofold manner; namely, in and out of the fit, in order to prevent that impression already alluded to as caused by repetition. 3. When we employ constraint as a remedy—and this may serve as a very general standard rule—a reaction of an inferior degree becomes weaker, and a reaction of a higher degree, stronger by repression; as a storm extinguishes a small fire, and fans a large one. Further: the more the psychical causality predominates, the sooner will the restraint and the succeeding reflex action avail; while, on the contrary, the physical operation of nature will take its headlong course. The *mania ephemera* expires, whether repressed or not, in and through its own attack. These regulations must be adhered to, but always individualized, to suit particular cases. A mistaken philanthropy has attempted, in recent times, entirely to do away with coercive measures, by the system of non-restraint. Theory and experience show that, as concerns the patient, they are as little to be dispensed with as instruments in surgery; and as concerns security, not without great danger. Let humanity be shown in the wisdom of their application.

"Constraint is effected psychically and physically: psychically, by the means indicated; but as respects solitude, with this caution, that it be applied to lunatics only temporarily, because, if protracted, it deadens the effect, heightens the mania, concentrates the moria into fixed delusion, and more rapidly changes both into idiocy. Physically, there are many measures in ordinary use, among which are the following:—

"*a.* Remedies which directly check emotion; the strait-jacket, the constraint-chair, the constraint-bed, bonds or shackles of every kind,—their application is justified by experience. They act by producing absolute depression of the disturbed mind, and are therefore indicated only *when and so long as they produce that effect.* They often act mechanically in preventing half voluntary motions; for instance, in the case of Onanists, who practise their ruinous habit by night, frequently without being conscious of it when half asleep. In patients who have been subjected to such restraint, the mere threat of repeating it is often sufficient to prevent a coming attack; others even call for it when they feel the approaching excitement, or when the fit is over, thank the physician—whom they avoid during its continuance—for the measures which he had adopted. The strait-jacket must be applied in slightest attacks, the constraint-chair in more severe cases. Ideler, however, advises that its application should not be continued longer than one or, at the very most, two days; for otherwise, œdematous swelling of the legs, erysipelas, proctocele, &c., are easily induced. When the mania is of longer duration, he, therefore, prefers the constraint-bed, recommended by Neumann. The strait-jacket possesses the advantage of causing no pain, no injurious friction, it is the least formidable in appearance, and does not impede the circulation, nor even motion, except that of the arms. The complete prevention of motion externally increases the patient's fury internally.

"*b.* Means which so shock the nervous system, that the motor innervation is extinguished. Among these are the swing, the revolving chair, the plunging bath; they must be used with due circumspection, and a cautious regard to contra-indicating conditions, such as a threatening of apoplexy, hemorrhages, &c. In this class, again, work must be placed—an indispensable means for acting on the nerves, even in this form of insanity. The experience of every psychological physician proves its applicability. Maniacs rave at their work, but they perform it, nevertheless. With respect to cold water, it is to be observed, that the too protracted and irrational use of it, in the form of douches, &c., has often caused a transition to incurable idiocy, as has been proved by finding, on post mortem examination, a softening of the brain. (The Graefenberg system, in particular, has furnished several candidates for lunatic asylums.) This observation is confirmed by multiplied experience.

"*c.* Remedies which excite pain, by a lesion of the skin. Ideler observes that, besides their main effect, they have also a very useful secondary influence over the hallucinations; he objects to the inunction of tartar emetic ointment on the head, on account of its slow operation, its inconvenience, and the abuse of it, for sometimes it is carried so far as to cause caries of the skull; while he commends the rapid and powerful effect of setons in the neck, and moxas on the spine; but prefers, above all, the electro-puncture, because it acts the most powerfully, and without occasioning a wound, or doing any harm. He mentions favourable instances of the employment of this remedy, and of urtication. It must never be forgotten, with respect to all these remedies, that pain, particularly if continued, and gradually increased to torture, is one of the most powerful means of weakening the whole organism.

"*d.* Remedies which so affect the nervous system, that excitement is impossible (metasyncrisis). To this head belongs treatment by nauseants and emetics, the sufficiently protracted application of which presents, it must be owned, many difficulties. Transitory mania is, however, for the most part, cured by *emeto-catharsis.* Opium, as a soporific, either fails altogether in producing sleep—since, in mania, as in tetanus, the largest doses are often taken without effect—or the patients are more raving than ever when they awake. The use of digitalis in increasing doses, duly regulated, is more frequently advantageous.

"2. The treatment during the attack is rather negative than positive. The patient must be prevented from injuring himself and others. Positive remedies, both depressing and derivative, as well psychical as physical, must be employed during the period of intermission, or shortly before the approaching fit. Yet many measures, as calmants of excitement, are also negative. The patient should be removed to an apartment, neither too light nor yet quite dark, for total darkness only tends to irritate; all causes of excitement should be withdrawn, all lively impressions avoided. Experience has shown that mild superintendence has a better effect than rigorous constraint. The patient must not be exasperated, but kept in awe by calm decision and equanimity, which overcome all resistance. A monotonous noise, soothing music, the view of a garden, of a peaceful, cheerful landscape, may tend to induce religious impressions, during the intervals, so as to reduce the temper of the patient to a normal state. I have myself witnessed the unexpectedly favourable effect which mild treatment produced on a maniac, when, after the lapse of many years, he was withdrawn from a rigorous system, under which he had frequently suffered chastisement. A sense of gratitude towards his deliverers overcame him, he was extremely collected in their presence, the fits were less frequent, and at length entirely ceased; but the mania became converted into moria.

"Somatically a local affection of the brain is frequently a cause, but oftener a consequence, of mania. This begets, through the orgasm caused by it, a secondary irritation of the brain, in consequence of which, when often repeated, or of long duration, plastic abnormities are formed, and become the proximate cause of the idiocy into which it so frequently passes. This is the moment when much may be effected towards the cure by means of blood-letting, partly general, partly local, which is never indicated in mania itself, but only in the hyperæmia superinduced by it. In cases of suppressed secretions (for instance, of the milk and of the lochia in puerperal mania, &c.), their restoration must be attended to. They are, however, not always causes, but also not unfrequently consequences of mania. We often see profluvia suppressed by violent emotions."—pp. 349—353.

We feel almost ashamed of the length of the above extract, but we have given it because it contains our author's views on several highly important points of treatment. Some of his statements are undoubtedly open to discussion, but, as we observed at the commencement of the article, it was our intention to give an *analytical* rather than a *critical* review. We proceed to give a condensed sketch of the further treatment of this form of insanity. When the prolonged irritation of the brain has caused a great depression of the functions of the nerves of nutrition, *tonico-solventia* are useful, and a cool regimen, water-drinking, cold baths, and ablution are serviceable. Cold shower-baths, applications of ice, &c., may likewise be employed when the patient is not in a fit. Solvents and aperients are, according to our author, almost always indicated alternately with nauseants. Of the much extolled narcotic remedies, the pungent narcotics, for instance, digitalis, are the only ones which deserve to be more generally employed.

With respect to diet, the lowering system has its limits, as was shown by Pinel's experience, who observed that the patients in the Bicêtre became more furious, and the mortality increased, when the famine during the revolution extended also to them. The diet, according to our author, must be mild and unstimulating, without amounting to a system of treatment by starvation. Tranquillity, sleep, and nourishment have cured many a maniac.

We regret that our allotted limits hinder us from touching upon the subjects of the treatment of idiocy, and the treatment during convalescence. The former of these subjects will probably be soon brought in a separate form before the readers of this journal.

We shall conclude our notice of the therapeutical portion of this treatise with the following sound remarks on the advantages of public institutions over private treatment:

"In one respect, private treatment would have an important advantage over public. If in any disorder it be important for the physician to have a thorough knowledge of the patient, of his personal character, of his connexions, of his position, accurately to know his history, carefully to observe him at all hours, and under all circumstances, to adapt the system of cure completely to his individual case, to devote his attention almost exclusively to him—in a word, to be the friend of the patient; and to live a part of his life with him, it is in a disorder of the relation between body and mind. This kind of ideal treatment could be carried out consistently only in private practice, on one patient at a time. But this ideal, like almost every other in life, must be renounced, because the other conditions under which alone cure is possible, can only be united in institutions, and, indeed, only in such as are public—that is, under the inspection of the state; for in private institutions lunatics are too much withdrawn from the eye of the authorities, independently of the greater difficulties of management and the expense.

"The chief grounds—partly negative, partly positive—for treatment in establishments, are the following:—

"1. The isolation of the patient, with a change of his situation, is the first condition, and the first step to every cure of mental diseases. It is not to be effected in the dwelling of the patient.

"2. Discipline and control are the most effectual aids to psychical treatment. These are impracticable in the residence of the patient.

"3. The lunatic asylum, as such, if it in any degree approximate to ideal perfection, is in itself a remedy, and many a patient has recovered without the application of any further means, merely by residing in it; nay, there are instances in which the very entrance into the establishment, without any other treatment whatever, has sufficed to rouse the patient out of his dream, just as the transitory state of intoxication is often shaken off by a sudden change of situation. It combines in itself the powers of restraint, domestic order, example, education, recreation, variety, &c.

"The disadvantages which public establishments bring with them, and which chiefly consist in the roughness of the attendants, and the difficulty of managing them in the mediate administration through non-medical men, and in the wound inflicted on the patient's sense of honour, may be avoided or neutralised. Perhaps the most difficult problem which they present, is how to maintain that incessant observation of the patient which is necessary to a knowledge of his state, and consequent treatment. It is a problem, however, which, through the co-operation of others, admits of being rendered more easy of solution to the physician, and which he must, after all, exert himself to execute to the best of his ability. Encouraging experience—nay, even the testimony of those who have been patients, sufficiently proves the beneficial effects of these institutions, even in their comparatively imperfect condition. Professor Exner saw a convalescent who, after being once cured, and feeling a return of the disorder, put the horses to his own carriage, and drove to the institution, which was several leagues distant."—pp. 359—361.

Of the appendix—on judicial psychology—we need say little. It is included in ten pages, and is of a more sketchy character than the earlier portions of the work. It embraces the consideration of the three following questions :—

1. The competency of medical psychology to bear a judicial application, whether in criminal or civil proceedings.
2. The notion of responsibility.
3. The detection of simulated or dissimulated psychical conditions.

To the two first of these questions we shall not on the present occasion refer. Considered in their several bearings they would occupy much more space than we could now devote to them; we shall, therefore, content ourselves with the following extract from our author's remarks on the third question :—

"The discovery of dissembled psychopathic states calls for as careful a regard as possible to all the circumstances connected with the anamnesis—such as the descent, education, mode of life and of thinking, &c. of the patient, together with a thorough examination of him, wherein all conceivable psycho-physical circumstances, the representation and import of which constitute the substance of medical psychology, must be fully investigated.

"The detection of simulated (feigned) psychopathies is likewise attended with great difficulties. The two objective somatically characteristic symptoms which Friedreich mentions as criteria of the psychoses—namely, the specific smile and the peculiar physiognomy—are by no means always present. The physiognomy may, moreover, be counterfeited so as to deceive. Even the sleeplessness generally (but not always) connected with psychopathies, may be assumed (though hardly beyond a certain point). The reluctance to look you in the face is not to be depended upon, because it also occurs in lunatics. In all cases, a knowledge of the object of the supposed simulation is of much importance, because it points out a motive through which (after many a contrivance to be invested according to the individual case) the mind of the supposed simulator may be acted upon. Moria and idiocy are more frequently, mania more rarely, simulated. In pretended monomania, the existence or non-existence of hereditary disposition, of physical symptoms, and of other (ethical) motives, for the imputed crime, in connexion with a comparison of the deed with the character of the individual, which must be inquired into in all attainable particulars, may serve as guides to the decision of the physician; wherein it is true, as in every decision, no small scope must be left to the judgment for a free exercise of its powers. Hypocrites, in general, adopt conduct diametrically opposite to their natural character: the cunning pretend to be stupid; the cheerful, melancholy; the gentle, furious, &c. Let the physician, therefore, set out from this point; let him accurately examine the harmony or want of harmony in the psychical and physical qualities of the individual; let him inquire long; surprise, deceive, tire the feigner; let him employ disagreeable psychical and physical means, as alleged remedies; with women, those which may disfigure them; and in the most obscure cases, let him await with an indifference, which is, beyond measure, painful to the simulator, the effects of time, the solver and elucidator of all things. It must not be forgotten, however, that men have really become mad after having been long confined on suspicion of pretending to be so, just as hysterical women often really fall into a state which they have long affected."—pp. 376—378.

And here we terminate our notice of this highly interesting and valuable work. Although in the practical treatment of insanity we believe that on the whole the English and Americans are at the present day the most successful physicians, we cannot close our eyes to the progress made by other nations in this question of humanity and science. Germany has done, and is still doing, her part in this noble—we may almost say, this divine—cause, and in the present volume she has found an able and an honest advocate.

